# Profound Hypoglycemia with Ecstasy Intoxication

**DOI:** 10.1155/2015/483153

**Published:** 2015-01-27

**Authors:** Perliveh Carrera, Vivek N. Iyer

**Affiliations:** Division of Pulmonary and Critical Care Medicine, Mayo Clinic, Rochester, MN 55905, USA

## Abstract

*Background.* 3,4-Methylenedioxymethamphetamine (MDMA) or ecstasy is a synthetic drug that is commonly abused for its stimulant and euphoric effects. Adverse MDMA effects include hyperthermia, psychomotor agitation, hemodynamic compromise, renal failure, hyponatremia, and coma. However, endogenous hyperinsulinemia with severe persistent hypoglycemia has not been reported with MDMA use. *Case Report.* We report the case of a 29-year-old woman who remained severely hypoglycemic requiring continuous intravenous infusion of high-dose dextrose solutions for more than 24 hours after MDMA intoxication. Serum insulin and C-peptide levels confirmed marked endogenous hyperinsulinemia as the cause of the severe hypoglycemia. *Why Should an Emergency Physician Be Aware of This?* Immediate and frequent monitoring of blood glucose should be instituted in patients presenting with MDMA ingestion particularly if found to be initially hypoglycemic. Early recognition can help prevent the deleterious effects of untreated hypoglycemia that can add to the morbidity from MDMA use. Clinicians need to be aware of this side effect of MDMA so they can carefully monitor and treat it, especially in patients presenting with altered mental status.

## 1. Introduction

3,4-Methylenedioxymethamphetamine (MDMA) commonly known as ecstasy is a synthetic drug used for its euphoric properties [1]. MDMA intoxication is known to cause hyperthermia, hepatotoxicity, psychomotor agitation, acute kidney injury, cardiovascular toxicity, hyponatremia, serotonin syndrome, and coma [1–4].

## 2. Case Report

A 29-year-old woman with depression and polysubstance abuse was taken to an outside hospital after being found on the floor minimally responsive. She had a history of daily cannabis and methamphetamine use along with prior suicide attempts. She was emergently intubated for airway protection. Initial labs revealed severe hypoglycemia with a glucose level of 20 mg/dL which increased to 62 mg/dL following an intravenous 50 mL bolus of 50% dextrose (D_50_W). The patient was subsequently transferred to our institution for management.

In the emergency room, she was again found to be severely hypoglycemic with a glucose level of 47 mg/dL. She received 100 mL of D_50_W intravenously which increased the glucose level to 101 mg/dL 30 minutes later. Prior to transfer to the intensive care unit (ICU), glucose level was rechecked and noted to be 37 mg/dL. Another 100 mL of D_50_W was administered and an infusion of normal saline with dextrose 10% (D_10_) at 100 mL/hr was started.

Vital signs in the ICU were as follows: temperature 36.4°C, heart rate 72 beats/min, blood pressure of 144/80 mmHg, and respiratory rate of 22 breaths/min on mechanical ventilation. Examination revealed a sedated and intubated patient with pinpoint pupils. Heart sounds were normal with coarse breath sounds, and extremities were well perfused. Abdominal exam was unrevealing. Urine toxicology screen was positive for tetrahydrocannabinol (THC), amphetamines, and MDMA. Drug assays for salicylates, acetaminophen, and alcohol levels were negative. Laboratory testing revealed normal chemistries except for hypokalemia (2.9 mmol/L) and a mildly elevated blood lactate level (2.46 mmol/L).

The patient was again noted to be hypoglycemic with a blood glucose of 27 mg/dL while on D_10_ at 100 mL/hr. Glucose monitoring was scheduled every 30 minutes and the IV infusion was switched to D_20_ at 200 mL/hr with D_50_W pushes as needed. Given the persistent hypoglycemia, history of polysubstance use, and suicidality, coingestion of sulfonylureas or possibly insulin administration was entertained and insulin and C-peptide levels were measured. The insulin level was markedly elevated at 456 mcIU/mL (reference range 2.6–24.9 mcIU/mL) and C-peptide level was 24.9 ng/mL (reference range 1.1–4.4) confirming endogenous hyperinsulin production rather than exogenous self-administration.

The patient was successfully extubated after 18 hours. She continued to receive D_20_ infusion at 200 mL/hr for another 12 hours before glucose levels stabilized and switched back to D_5_W for maintenance. Repeat insulin and C-peptide levels 24 hours later were substantially lower at 19.9 mcIU/mL and 4.8 ng/mL, respectively. She made a full recovery without neurologic deficits and was transferred to the inpatient psychiatry unit on day 3.

## 3. Discussion

Severe persistent hypoglycemia with endogenous hyperinsulinemia due to ecstasy intoxication has not been previously reported in humans. In reviewing the literature, there is one case report that associated MDMA use with an episode of hypoglycemia which resolved after a one-time administration of 50 mL of D_50_W [5]. However, no insulin or C-peptide levels were reported as part of that report. There is data from one animal study that described the acute effects of MDMA on blood glucose levels in vivo. The study showed that, compared to controls, blood glucose levels in the experimental group dropped by as much as 54.4 ± 25.3 mg/dL 1 hour after a single dose of MDMA [6].

In addition to MDMA, our patient tested positive for 3 tetrahydrocannabinol (THC) and amphetamines. In our review of the literature, amphetamines and THC have not been associated with endogenous hyperinsulinemia related hypoglycemia. Interestingly, THC has been shown to suppress growth hormone and cortisol responses to hypoglycemia and this may have potentially prolonged the duration of hypoglycemia in our patient [7].

According to the 2012 National Survey on Drug Use and Health, approximately 13 million adults aged 18 to 25 reported ecstasy use at least once in their lifetime. In 2012, there were 869,000 new users of ecstasy aged 12 and older in the USA [8]. In addition, from 2005 to 2011, ecstasy-related US hospital and ED visits rose by 128%. Given the lethal effects of undetected hypoglycemia, healthcare providers must be very vigilant of severe and persistent hypoglycemia as a potential adverse effect of MDMA intoxication [9, 10].

In conclusion, we report the first case of documented endogenous hyperinsulinemia and persistent hypoglycemia associated with MDMA intoxication. We recommend frequent blood glucose monitoring and aggressive correction of hypoglycemia especially in obtunded patients.
